# Region-informed machine learning model for choroid plexus segmentation in Alzheimer’s disease

**DOI:** 10.3389/fnagi.2025.1613320

**Published:** 2025-07-23

**Authors:** Liangdong Zhou, Tracy A. Butler, Xiuyuan H. Wang, Samantha A. Keil, Seyed Hani Hojjati, Tsung-Wei Hu, Mélissa Savard, Firoza Z. Lussier, Pedro Rosa-Neto, Lidia Glodzik, Mony J. de Leon, Gloria C. Chiang, Yi Li

**Affiliations:** ^1^Department of Radiology, Brain Health Imaging Institute, Weill Cornell Medicine, New York, NY, United States; ^2^Douglas Hospital Research Centre, Douglas Mental Health University Institute, Montreal, QC, Canada; ^3^Department of Psychiatry, University of Pittsburgh, Pittsburgh, PA, United States; ^4^Departments of Neurology and Neurosurgery and Psychiatry, McGill University, Montreal, QC, Canada

**Keywords:** choroid plexus, Alzheimer’s disease, cerebrospinal fluid, neurofluids, brain clearance, Gaussian mixture model, FreeSurfer

## Abstract

**Introduction:**

The choroid plexus (CP), a critical structure for cerebrospinal fluid (CSF) production, has been increasingly recognized for its involvement in Alzheimer’s disease (AD). Accurate segmentation of CP from magnetic resonance imaging (MRI) remains challenging due to its irregular shape, variable MR signal, and proximity to the lateral ventricles. This study aimed to develop and evaluate a region-informed Gaussian Mixture Model (One-GMM) for automatic CP segmentation using anatomical priors derived from FreeSurfer (FS) software and compare it with manual, FS, and one previous GMM-based (Two-GMM) methods.

**Materials and methods:**

T1-weighted (T1w) and T2-fluid-attenuated inversion recovery (FLAIR) MRI scans were acquired from 38 participants [19 cognitively normal (CN)], 11 with mild cognitive impairment (MCI), and 8 with AD. Manual segmentations served as ground truth. A GMM was applied within an anatomically constrained region combining the lateral ventricles and CP derived from FS reconstruction. The segmentation accuracy was assessed using the dice similarity coefficient (DSC), the 95th percentile Hausdorff distance (HD95), and volume difference percentage (VD%). Results were compared with those from FS and one previous GMM method-based segmentations across diagnostic groups.

**Results:**

The region-informed One-GMM achieved significantly higher accuracy compared to FS and Two-GMM, with a mean DSC of 0.82 ± 0.05 for One-GMM versus 0.24 ± 0.11 for FS (*p*  <  0.001), and 0.66 ± 0.10 for Two-GMM (*p* < 0.001), lower HD95 (One-GMM: 6.06 ± 10.32 mm vs. FS: 26.21 ± 7.32 mm vs. Two-GMM: 10.58 ± 6.47 mm), and comparable volume difference (One-GMM: 20.97 ± 9.53% vs. FS: 24.32 ± 28.13% vs. Two-GMM: 24.27 ± 22.10, *p* = 0.87). Segmentation accuracy of our proposed method was consistent across all diagnostic groups. Clinical analysis showed that there is no diagnostic group difference in CP volume obtained from manual, FS, Two-GMM, and our proposed One-GMM methods. In the whole cohort, there are also no age and sex effects of CP volume with all methods. Restricting to the CN group, CP volume from both manual (*p* = 0.03), Two-GMM (*p* < 0.01) and the proposed One-GMM (*p* = 0.05), methods show an aging effect, but not for the FS segmented CP volume (*p* = 0.22).

**Conclusion:**

A region-informed One-GMM method significantly improved CP segmentation accuracy over FS, providing a practical and accessible tool for CP quantification in AD and other research studies. Within this small cohort, no diagnostic group difference in CP volume was observed. An aging effect of CP volume was found within the CN group.

## 1 Introduction

The choroid plexus (CP), a part of the brain located in the lateral ventricles (LV), is responsible for producing cerebrospinal fluid (CSF) and maintaining the blood-CSF barrier (BCSFB) (Čarna et al., 2023; Cserr, 1971). Beyond these fundamental roles, the CP has garnered significant attention in neurodegenerative research due to its involvement in immune surveillance (Dumas et al., 2024; Xu et al., 2024), clearance of metabolic waste (Bergsland et al., 2024), and regulation of neuroinflammation (Althubaity et al., 2022). Emerging evidence suggests that alterations in CP structure and function are closely linked to the pathophysiology of Alzheimer’s disease (AD), highlighting its potential as a biomarker for disease progression and severity (Lu et al., 2023; Butler et al., 2023; Althubaity et al., 2022).

Recent studies have demonstrated that increased CP volume correlates with greater cognitive impairment in individuals on the AD spectrum (Hidaka et al., 2024; Jeong et al., 2025). For instance, it has been demonstrated that patients with AD exhibited significantly larger CP volumes compared to healthy controls, with volumes inversely related to Mini-Mental State Examination (MMSE) scores (Čarna et al., 2023). Similarly, another study found that among patients with cognitive symptoms, larger CP volumes were associated with more severe cognitive deficits, independent of beta-amyloid (Aβ) pathology or neurodegeneration (Jeong et al., 2025; Choi et al., 2022). These findings underscore the importance of accurately quantifying CP volume to better understand its role in AD progression, to potentially serve as an imaging biomarker for cognitive decline, and to facilitate accurate regional functional measures like blood flow and permeability (Bouzerar et al., 2013; Tadayon et al., 2020).

Despite the recognized significance of CP volume in AD research, precise and automated segmentation of the CP remains a formidable challenge (Lu et al., 2023; Li et al., 2024). The CP’s irregular morphology, proximity to the wall of lateral ventricles, and subtle contrast differences from surrounding tissues on standard T1-weighted (T1w) MRI scans complicate its delineation. Traditional neuroimaging tools, such as FreeSurfer (FS) (Fischl, 2012), have been employed to segment the CP; however, studies indicate that FS automatic segmentations often lack the accuracy and reliability required for clinical and research applications (Storelli et al., 2024). Manual segmentation, while considered the gold standard, is labor-intensive, subject to inter-rater variability, and impractical for large-scale studies (Hidaka et al., 2024; Bannai et al., 2023).

To address these challenges, machine learning (ML) and deep learning (DL) based approaches have been explored to enhance CP segmentation (Storelli et al., 2024; Tadayon et al., 2020; Zhao et al., 2020; Eisma et al., 2024). Among these, the Gaussian Mixture Model (GMM) has shown promise (Tadayon et al., 2020). GMM is a probabilistic model that assumes all data points are generated from a mixture of several Gaussian distributions with unknown parameters. In the context of CP segmentation, GMM can effectively model the intensity distributions of different tissue types, facilitating the differentiation of the CP from adjacent structures and CSF. A study demonstrated that two-stage GMM-based segmentation outperformed the FS automatic method and showed high similarity with manual segmentation, with Dice similarity coefficient (DSC) ranging from 0.55 to 0.73 across multiple datasets, providing more accurate and reliable CP delineations (Tadayon et al., 2020). However, these DSC ranges of currently reported ML and DL-based segmentation have yet to reach the levels of accuracy required for broad clinical applications.

Incorporating anatomical priors into ML models can further enhance segmentation accuracy. By integrating region-specific information, such as the combined volume of the lateral ventricles and CP obtained from FS reconstruction, the model can be guided to focus on areas where the CP is anatomically expected.

In this study, we propose a region-informed GMM for the automatic segmentation of the CP in individuals with Alzheimer’s disease. By utilizing the combined LV and CP regions segmented from FS reconstruction as anatomical priors, our model aims to enhance segmentation accuracy and reliability. We hypothesize that this approach will outperform traditional methods, such as FS-based CP segmentation, providing a valuable tool for investigating the role of CP volume in AD research, including brain clearance, glymphatic function, and neurofluid dynamics (Eide et al., 2020).

## 2 Materials and methods

### 2.1 Participants

All participants were recruited between 2018 and 2019 as part of the Translational Biomarkers of Aging and Dementia (TRIAD) study conducted at McGill University. Subjects consisted of 19 cognitively normal (CN) controls, 11 mild cognitive impairment (MCI), and 8 subjects diagnosed with AD by a physician using accepted criteria. MCI and AD subjects had a positive Aβ PET scan and a Cognitive Dementia Rating Scale (CDR score between 0.5 and 2). CN subjects had no objective cognitive impairment and a CDR score of 0. All subjects were free from significant medical or neurologic disease (other than AD). The study was approved by the Douglas Mental Health University Institute Research Ethics Board, and written informed consent was obtained from all participants.

### 2.2 MRI data acquisition

Three-dimensional volumetric T1-weighted (T1w) MPRAGE were acquired using a Siemens Skyra 3 T scanner with acquisition parameters: repetition time (TR) = 2,300 ms, echo time (TE) = 2.96 ms, flip angle = 9^o^, and voxel size 1 × 1 × 1 mm^3^. T2-FLAIR images were acquired in the same session with an isotropic voxel size of 1 × 1 × 1 mm^3^ for all participants. Details of the data acquisition were documented in the previous literature (Butler et al., 2024; Pascoal et al., 2021; Macedo et al., 2024).

### 2.3 Image preprocessing

All MRI scans underwent a standardized preprocessing pipeline in FreeSurfer v7.1 using the *recon-all* command, which processes T1w into a FS space with standard regions of interest (ROI) parcellations. Specifically, FS output includes the segmentation of lateral ventricles (LV) and CP, providing anatomical priors for the subsequent segmentation process. The accuracy of the LV mask from FS reconstruction was screened by making a snapshot of the LV mask overlaying T1w, and necessary manual edits were performed to make the LV mask accurate. The FS segmented CP was also used to compare with our proposed method. T2-FLAIR is processed with N3 correction to remove the regional brightness inhomogeneity (Tustison et al., 2010), and coregistered to FS T1w space.

### 2.4 Manual segmentation of CP

The manual segmentation of CP was performed on the T2-FLAIR image in the FS T1w space by drawing the bright voxels located in the lateral ventricles and verified against the T1w scan for anatomical accuracy. This process was conducted by a trained radiologist, Dr. Savard, and re-examined by Dr. Lussier to guarantee the accuracy and consistency.

### 2.5 Region-informed Gaussian mixture model for CP segmentation

**Figure 1:**
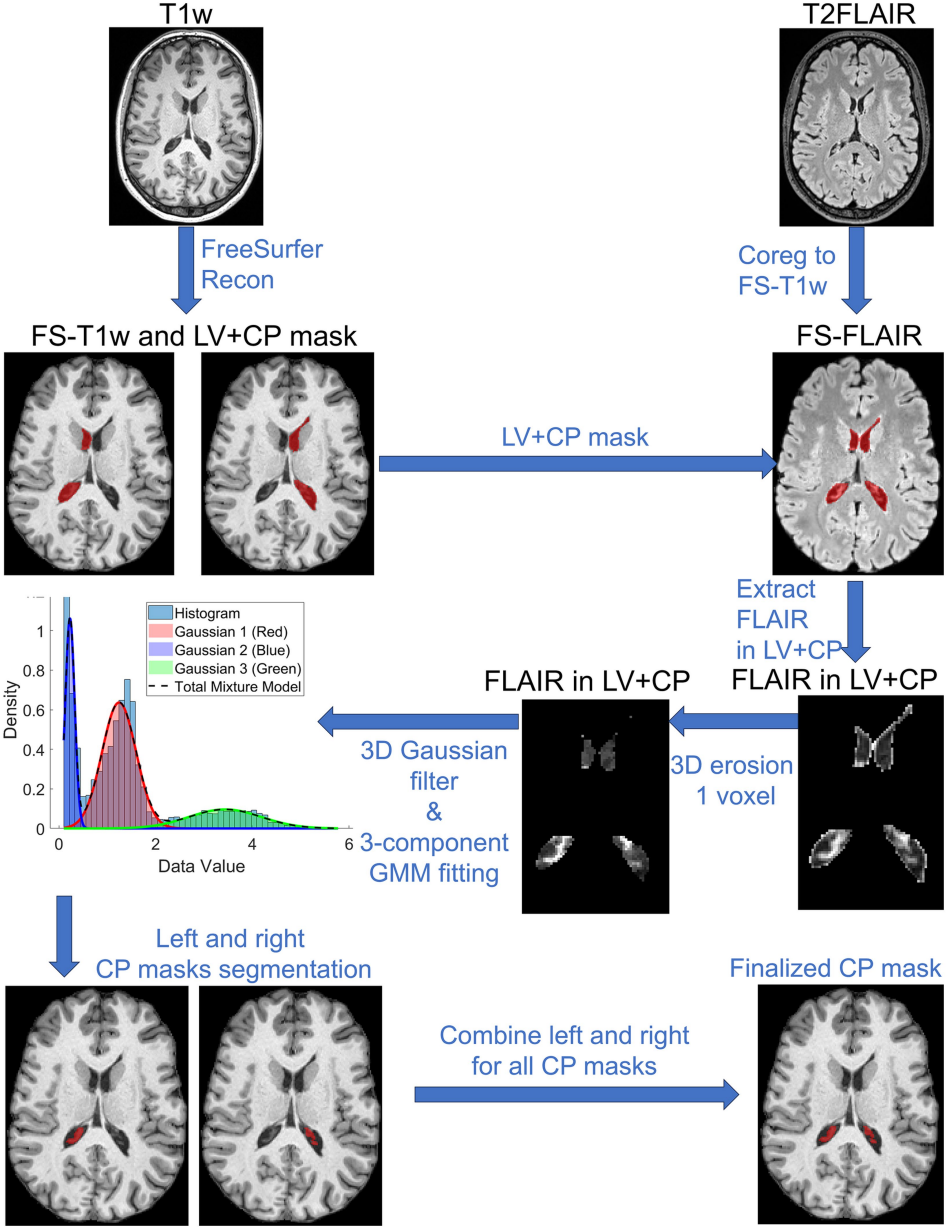
Flowchart of the choroid plexus segmentation using Gaussian Mixture Model with FreeSurfer processed lateral ventricles and choroid plexus priors. T1w, T1-weighted image; T2FLAIR, T2-weighted fluid attenuation image recovery; FS, FreeSurfer; LV, lateral ventricle; CP, choroid plexus; Recon, reconstruction; Coreg, coregistration.

Our proposed segmentation approach utilizes a Gaussian Mixture Model (GMM) informed by FS-segmented LV + CP. Specifically, to apply GMM, the T2-FLAIR images in the LV + CP region, eroded by one voxel using a sphere kernel, were applied a 3D Gaussian filter to smooth the intensity of voxels, followed by a normalization step that divided the image values by the non-zero voxel mean. A 3-component GMM model was applied to the result image with our empirical initial mean values μ=[0.15,1.5,4] and a corresponding standard deviation (SD) σ=[0.02,0.1,1.5] with component proportion [0.45,0.5,0.05] . Note that these initial means and standard deviations were obtained from testing on typical cases, and they are not fixed and can easily be adapted for a new dataset by testing on several cases. The GMM assigns each voxel a probability of belonging to the CP or the other two components based on its intensity and spatial location. The component/group that has the highest mean image intensity was assigned as the voxels in CP based on the posterior probability calculation and hard clustering rule, that is, assigns voxels with the highest posterior probability to the CP region. All these processing steps were conducted separately on the left and right sides to avoid the effect of the size variation of CP between hemispheres. The detailed steps of the processing were shown in the flowchart in Figure 1. This region-informed approach guides the GMM to focus on the areas where the CP is anatomically expected, enhancing segmentation accuracy. The implementation code of our proposed One-GMM method, written in MATLAB, will be made available as indicated in the “Data Availability” section.

### 2.6 Accuracy estimation of the choroid plexus segmentation using GMM

We compared our region-informed GMM (One-GMM) segmented CP to the FS’s automatic CP segmentation and manual segmentation, as well as the previously reported two-stage GMM (Two-GMM) method (Tadayon et al., 2020), using the following three metrics.

1. Dice similarity coefficient (DSC) (Zou et al., 2004), with formula DSC = 2TP/(2TP + FP + FN), where TP is the true positive, FP the false-positive, and FN the false-negative. DSC is to measure the overlap between the automatic and the manual CP segmentations, the higher the better.
2. Hausdorff distance with 95 percentiles (HD95) (Huttenlocher et al., 1993), to measure the maximum distance between the automatic and manual segmentation boundaries, the lower the better; and.
3. Volume difference percentage (VD%) (Taha and Hanbury, 2015), with formula VD% = (V_pred_-V_true_)/V_true_, where V_pred_ and V_true_ are predicted volume and ground truth volume, respectively. VD% is to estimate the bias in total volume regardless of shape or location; the lower the better. Since the CP is small, irregular, and often adjacent to ventricles, the combination of these three metrics helps us to evaluate the accuracy of segmentation comprehensively.

### 2.7 Statistical analysis

All statistical analyses were performed in R v4.4.3 within RStudio v2022.07.1. We used the Kruskal–Wallis Rank Sum Test with *post hoc* Dunn’s test of multiple comparisons following a significant Kruskal-Wallis Test for assessing the diagnostic group differences in segmentation accuracy. The impact of diagnosis (CN, MCI, and AD) on model performance was also examined using Tukey’s Honestly Significant Difference (HSD) test. The paired Wilcoxon Signed Rank Test was used to compare the performance of our proposed One-GMM method with the FS method, as well as the previous Two-GMM method, and it was also used to compare the segmented CP volume using the proposed One-GMM, FS, and previous Two-GMM with manual segmentation. A clinical analysis was conducted to evaluate the diagnostic group difference of segmented CP volume with the Kruskal–Wallis Rank Sum Test. The age and sex effects of segmented CP volume were modeled using linear regression. Statistical significance was set at *p* < 0.05, and all *p*-values were adjusted for multiple comparisons when necessary.

## 3 Results

### 3.1 Demographics and measurements

Among the total of 40 participants, there were 19 CN, 11 MCI, and 8 AD. Their age, sex, and CP segmentation-related measurements are listed in Table 1. No group differences were found for age, sex, or CP volume from all methods. All methods show no performance difference across diagnostic groups. We observed that the proposed region-informed GMM model outperformed the other methods in all three metrics—DSC, HD95, and VD%—among all diagnostic groups compared with the FS and Two-GMM methods.

**Table 1:** Demographics and measurements.

Items	CN	MCI	AD	*p*-value
Subjects number (*n*)	19	11	8	
Age: Years [mean (SD)]	72.70 (4.77)	71.63 (6.17)	70.99 (7.80)	0.57
Sex: M (%)	5 (26.3)	8 (72.7)	3 (37.5)	0.07
CP volume manual [ml, mean (SD)]	1.77 (0.85)	1.94 (0.35)	2.06 (0.91)	0.55
CP volume proposed One-GMM [ml, mean (SD)]	1.39 (0.67)	1.53 (0.41)	1.79 (0.82)	0.40
CP volume Two-GMM [ml, mean (SD)]	1.76 (0.72)	2.41 (1.47)	2.38 (0.87)	0.28
CP volume FS [mean (SD)]	1.61 (0.51)	1.83 (0.54)	2.06 (0.60)	0.16
DSC proposed One-GMM [mean (SD)]	**0.83 (0.04)**	**0.81 (0.05)**	**0.82 (0.06)**	0.70
DSC FS [mean (SD)]	0.20 (0.08)	0.25 (0.12)	0.31 (0.11)	0.06
DSC Two-GMM [mean (SD)]	0.67 (0.10)	0.65 (0.11)	0.67 (0.10)	0.91
HD95 proposed One-GMM [mean (SD)]	**3.46 (8.40)**	**7.72 (11.34)**	**9.95 (12.58)**	0.26
HD95 FS [mean (SD)]	26.56 (5.03)	23.17 (7.64)	29.58 (10.36)	0.16
HD95 Two-GMM [mean (SD)]	9.20 (5.51)	11.11 (7.10)	13.16 (7.61)	0.32
VD% proposed One-GMM [mean (SD)]	**21.25 (7.56)**	**20.82 (11.83)**	**19.47 (10.97)**	0.92
VD% FS [mean (SD)]	25.98 (30.85)	23.40 (21.78)	28.96 (37.86)	0.90
VD% Two-GMM [mean (SD)]	20.40 (16.90)	24.22 (24.87)	33.51 (28.73)	0.36

GMM, Gaussian mixture model; CN, cognitively normal; MCI, mild cognitive impairment; AD, Alzheimer’s disease; CP, choroid plexus; FS, FreeSurfer; SD, standard deviation; DSC, dice similarity coefficient; HD95, Hausdorff distance at 95 percentiles; VD%, volume difference percentage; M, male. Bold style numbers indicate the best performance compared with other two methods.

### 3.2 Choroid plexus segmentations—visual comparison

**Figure 2:**
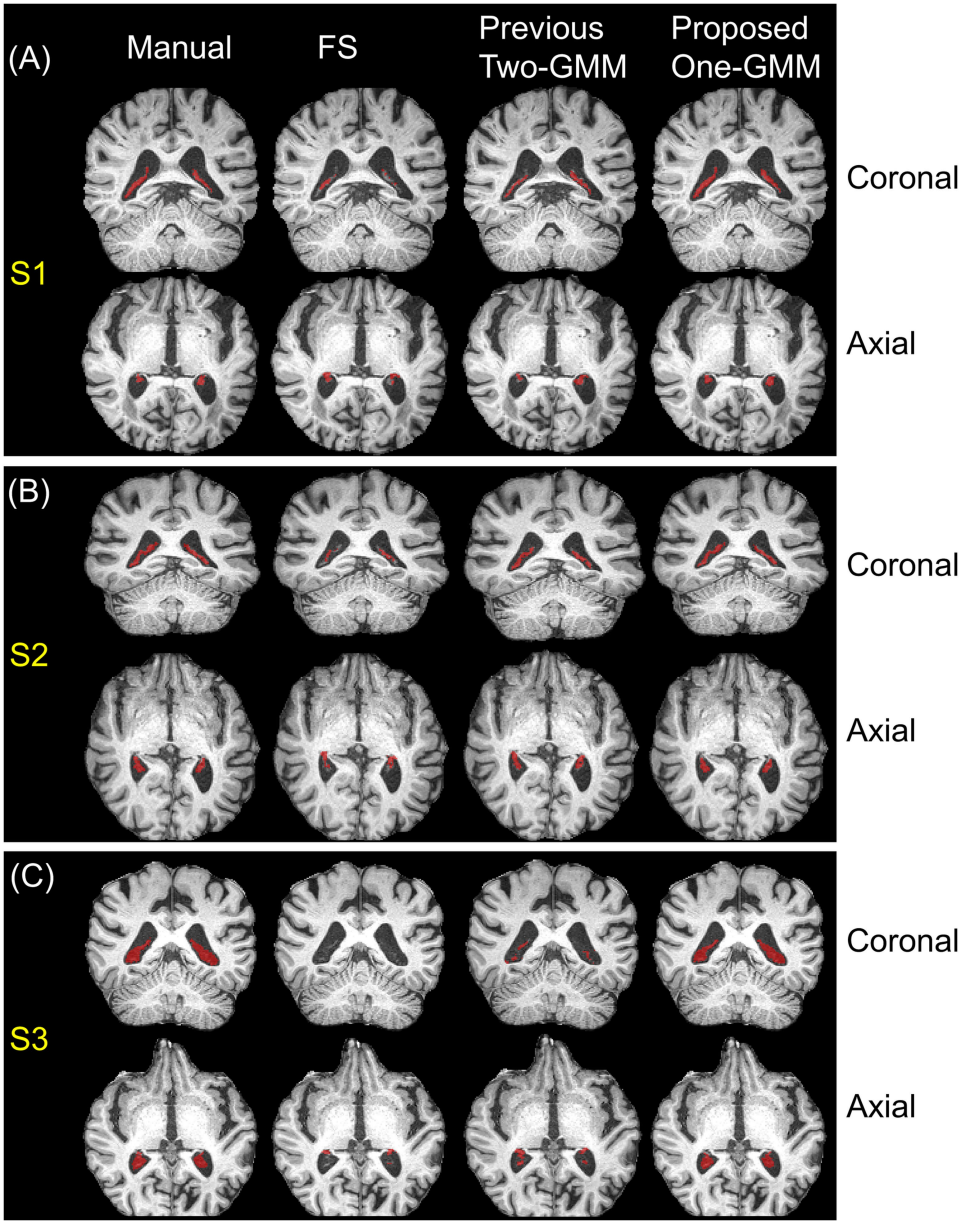
Examples of choroid plexus segmentations using manual (first column), FS reconstruction (second column), previous Two-GMM (third column), and our proposed region-informed One-GMM (fourth column) methods. **(A)** A subject S1 with low CP volume; **(B)** A subject S2 with medium CP volume; **(C)** A subject S3 with high CP volume. We can see that the proposed One-GMM method aligns well with manual segmentation and outperforms both FS and previous Two-GMM methods across subjects with different CP volumes. CP, choroid plexus; FS, FreeSurfer; GMM, Gaussian Mixture Model.

Figure 2 shows the examples of three participants (S1, S2, and S3) with varying CP volumes in both axial and coronal views. Figures 2A,C has small (S1), medium (S2), and high (S3) CP volume, respectively. We see that our proposed region-informed One-GMM method gives CP segmentation highly agrees with manually drawn ROIs and outperforms FS and previous Two-GMM methods, whose quantitative comparisons are discussed in the following two subsections.

### 3.3 Choroid plexus segmented volume comparison

**Figure 3:**
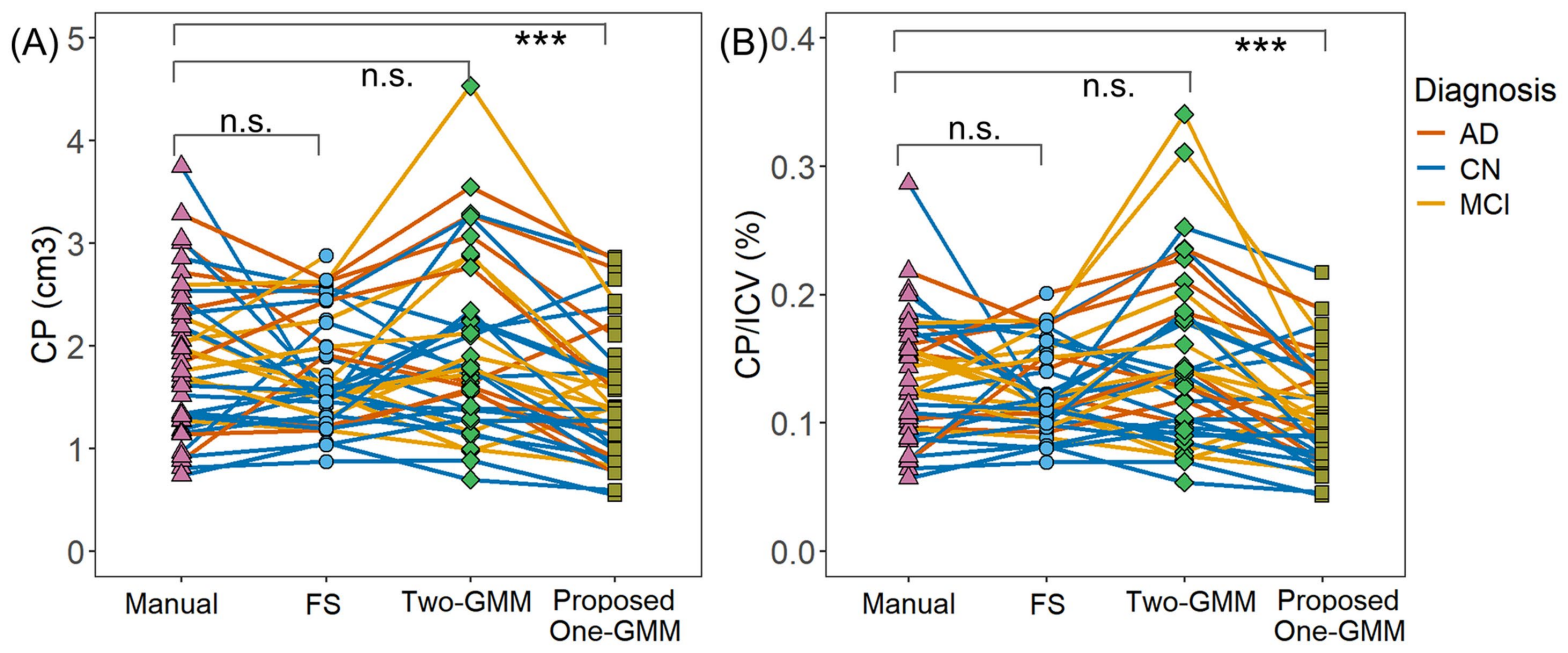
Segmented choroid plexus volume comparison across methods. **(A)** Choroid plexus volume comparison; **(B)** percentage of choroid plexus volume normalized by intracranial volume. The data showed that the CP segmentation by FS and Two-GMM methods was much deviated from the manual for many participants. Our proposed One-GMM method has a slight underestimation of CP volume, which might be due to the erosion process for removing the boundary voxels. Since the boundary voxels are prone to have partial volume effects, our erosion process is beneficial to exclude false-positive candidates. CP, Choroid plexus; ICV, intracranial volume. CN, cognitively normal; MCI, mild cognitive impairment; AD, Alzheimer’s disease; n.s., non-significant; ***p < 0.001.

The paired Wilcoxon Signed Rank Test showed that our proposed region-informed One-GMM method gave consistently slight underestimation of CP volume (V = 11, *p* < 0.001) compared with manual ground truth, while FS and Two-GMM methods show no over- or under-estimation of CP volume (FS: V = 315, *p* = 0.21; Two-GMM: V = 456, *p* = 0.22), as presented in Figure 3A. These findings have no change after accounting for the intracranial volume (ICV), the proposed One-GMM method (V = 14, *p* < 0.001), FS method (V = 324, *p* = 0.25), and the previous Two-GMM method (V = 458, *p* = 0.21), as presented in Figure 3B. Note that the segmentation of CP volume agreement between FS/Two-GMM and manual methods does not mean that the FS/Two-GMM segmentations are accurate. On the contrary, we can see in Figure 3 that CP volumes from FS/Two-GMM methods have both severely underestimation and overestimation, showing their inconsistent performance across participants. The error of CP segmentation from FS/Two-GMM methods is also seen in the second and third columns of Figure 2.

### 3.4 Choroid plexus segmentation accuracy

**Figure 4:**
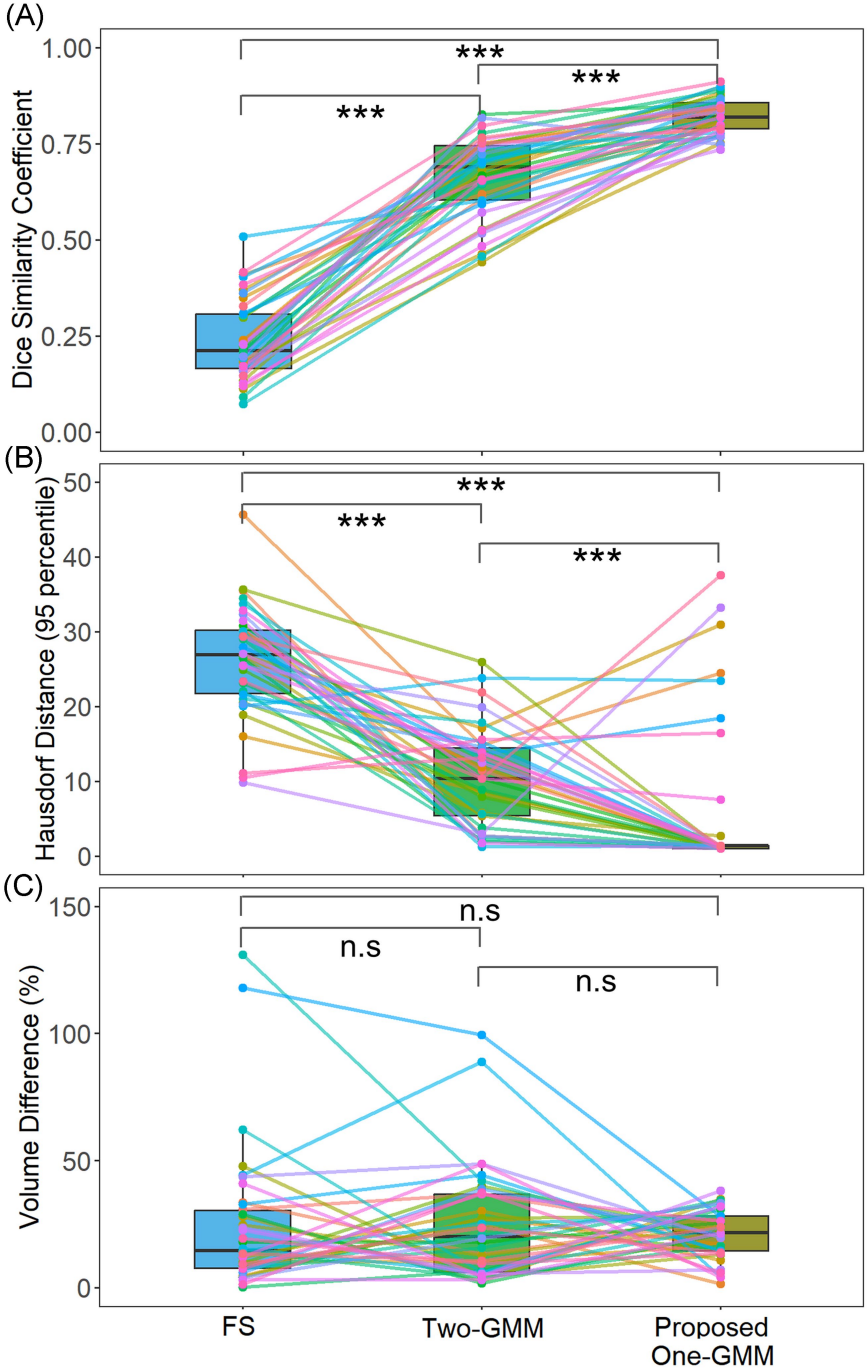
Accuracy of choroid plexus segmentation using FS, previous Two-GMM, and our proposed region-informed One-GMM methods. **(A)** Dice similarity coefficient; **(B)** Hausdorff distance at 95 percentiles; **(C)** Volume difference percentage. The results show that our proposed method has better DSC and HD95, while VD% is similar for all methods. Note that each dot and line in the boxplots represents a participant. DSC, Dice similarity coefficient; HD95, Hausdorff distance at 95 percentiles; VD%, volume difference percentage. FS, FreeSurferr; n.s., non-significant; ***p < 0.001.

Our region-informed Gaussian Mixture Model (One-GMM) achieved a mean Dice similarity coefficient (DSC) of 0.82 ± 0.04, significantly outperforming (*p* < 0.001) FS’s automatic CP segmentation with a mean DSC of 0.24 ± 0.11 and the previous Two-GMM method with DSC of 0.66 ± 0.10, as shown in Figure 4A. These results suggest that approximately 82% segmented voxels overlay with manually drawn ROI using our proposed One-GMM method. Additionally, our model demonstrated lower Hausdorff distances (Figure 4B, One-GMM: 6.06 ± 10.32 mm, FS: 26.21 ± 7.32 mm, Two-GMM: 10.58 ± 10.32 mm, all *p* < 0.001), indicating that our proposed method has better segmentation boundary consistency with the manual method. Moreover, our proposed method has a similar volume difference error (Figure 4C, One-GMM: 20.97 ± 9.53, FS: 24.32 ± 28.13, Two-GMM: 24.27 ± 22.10, all *p* > 0.05). In summary, all three typical measures for segmentation accuracy showed that our proposed method agrees with manual segmentation well and has superior performance than the FS/Two-GMM methods in the whole cohort and across diagnostic groups.

### 3.5 Clinical results

**Figure 5:**
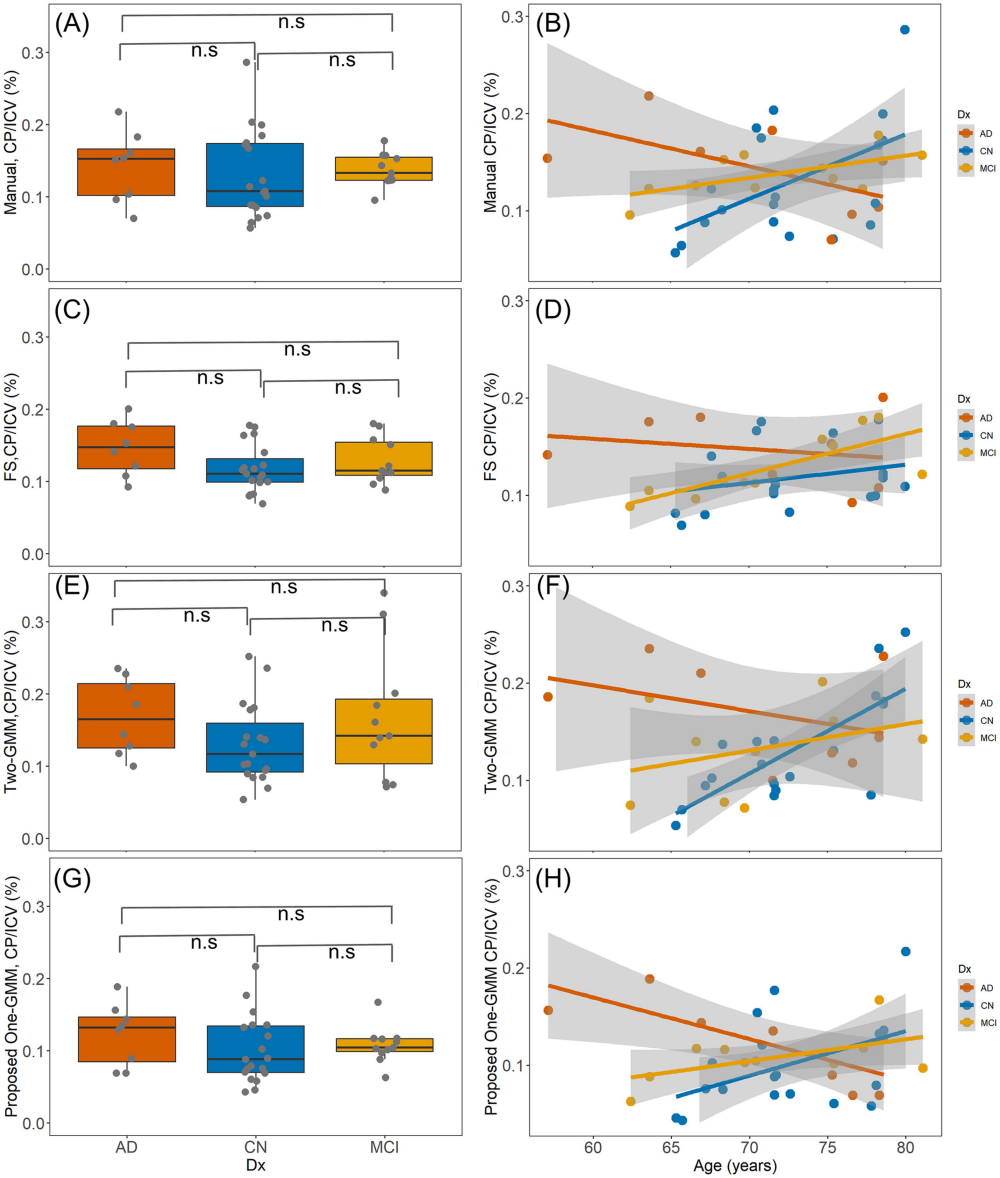
The clinical results of segmented CP volume using three methods. **(A,C,E,G)**, are the diagnostic group differences of CP volume from manual, FS, previous Two-GMM, and the proposed One-GMM methods, respectively. No diagnostic group difference of CP volume was observed from all these methods. **(B,D,F,H)**, are the age effects of CP volume from the three methods. In the whole cohort, no age effect of CP volume was observed for all methods. Restricting to the CN group, a positive association between age and CP volume from manual, previous Two-GMM, and the proposed One-GMM methods was observed, but not in the FS segmented CP volume.

The Kruskal–Wallis rank sum test showed that in this small cohort there is no diagnostic group difference of CP volume from all four methods (*p* > 0.05) including manual, FS, the previous Two-GMM, and the proposed One-GMM methods, as shown in Figures 5C–E,G, respectively. In the whole cohort, the linear regression analysis with CP volume as output and age and sex as independent factors showed that the CP volume has no age or sex effect. Restricting to CN group, we observed a normal aging effect of CP volume from manual (*t* = 2.41, *p* = 0.02, *R*^2^ = 0.18; Figure 5B), the previous Two-GMM (*t* = 4.63, *p* < 0.01; Figure 5F), and the proposed method (*t* = 2.05, *p* = 0.05, *R*^2^ = 0.12; Figure 5H) show CP volume increases with normal aging, but not saw the same results from FS method-based CP volume (*t* = 1.29, *p* = 0.22, *R*^2^ = 0.01; Figure 5D). No sex effect of CP volume was observed from all methods.

## 4 Discussion

This study presents a novel region-informed Gaussian Mixture Model for choroid plexus segmentation, which gives more accurate CP segmentation than previous studies and offers a promising approach for brain clearance studies in Alzheimer’s disease research. Our findings demonstrate that incorporating anatomical priors (lateral ventricles + choroid plexus) into the GMM framework substantially enhances segmentation accuracy, regardless of diagnostic groups. Clinical analysis within the cohort showed that CP volume, as determined by our proposed method, increased with normal aging and showed no group difference between the diagnostic groups of CN, MCI, and AD.

### 4.1 Clinical and research implications

Accurate segmentation of the CP is critical for volumetric/structural and functional analyses and understanding its role in neurodegeneration (Bergsland et al., 2024; Lu et al., 2023; Butler et al., 2023; Hidaka et al., 2024). Our data showed that the CP volume from the manual and proposed methods has a normal aging effect, which is consistent with previous studies (Eisma et al., 2024; Sun et al., 2024), while the FS-based CP volume has no such effect. Although we did not observe the diagnostic group difference in CP volume, which has been reported before (Choi et al., 2022; Jiang et al., 2024), we think this might be due to the small sample size. Another reason for these negative findings here probably arises from the fact that the anatomic change of CP might be later than its functional change (Gião et al., 2022; Delvenne et al., 2024). For instance, a recent study demonstrated that there is no group difference in CP volume between amyloid-positive and negative groups, but there is a group difference in their diffusion tensor imaging-based free water fraction (DTI-FWf) (Xu et al., 2025). Given that our proposed method produces more accurate CP segmentation with a DSC score of approximately 0.82, the group difference might be observed in a larger cohort using our proposed method. The improved precision offered by our model facilitates more reliable investigations into CP-related biomarkers, such as CP volume relative to intracranial volume or other function-related CP inflammation signals (Althubaity et al., 2022; Butler et al., 2023). This is particularly relevant in AD, where subtle changes in CP volume and structure may reflect disease progression or therapeutic response, and its volume alterations in might implicate mechanistic and functional impairment (Cserr, 1971; Althubaity et al., 2022; Choi et al., 2022; Bouhrara et al., 2024; Bouhrara et al., 2024; Rmeily-Haddad et al., 2011). CP is the key region that produces CSF in the brain, which plays crucial roles in protecting the central nervous system (CNS) and is widely considered as the neurofluid in the glymphatic system that works to remove metabolic wastes, including Aβ and tau proteins in AD (Zhou et al., 2024; Agarwal et al., 2024; Iliff et al., 2012; Ozsahin et al., 2025). CP volume changes may be associated with functional alterations, including decreased CSF production and/or brain clearance (Li et al., 2022; Zhou et al., 2024). Accurate assessment of CP volume will facilitate improved understanding of the role of CP in brain fluid clearance and other key functions, such as regulation of neuroinflammation, critical to the pathophysiology of neurodegenerative diseases like AD.

### 4.2 Lateral ventricle segmentation from FreeSurfer

Our proposed CP segmentation method depends on the LV mask from FS reconstruction. While it has been reported that FS v4.5-based LV segmentation for elder and Alzheimer’s disease subjects has an 11% failure rate (Kempton et al., 2011), due to significantly enlarged LV size. In this study, we used FS v7.1, which performs better than its previous versions. In an elderly cohort of 623 participants at the Brain Health Imaging Institute of Weill Cornell Medicine, we found that there are 35 (35/623 = 5.6%) participants whose FS reconstruction has errors in LV segmentation. However, we found none of those 35 failed LV segmentations affects the CP segmentation using our proposed One-GMM method, since the failure of LV segmentation generally underestimates the volume of CP, and the missing parts are usually superior slices of LV and sometimes small regions in inferior LV that are away from CP. Even though we still recommend carefully checking LV segmentation accuracy by taking snapshots of the LV mask overlay on T1w or T2w images. The FS segmented LV and CP masks are adjacent to each other, after merging them there is no gap in between and the erosion steps in our method will only remove voxels at the outer boundary of the merged mask and will not generate gaps between CP and LV, facilitating an accurate segmentation of CP in the following steps.

### 4.3 Comparison to existing methods

Our model outperforms FreeSurfer and previous Two-GMM approaches in DSC by integrating regional information that directs the model’s focus to anatomically plausible regions (Storelli et al., 2024; Tadayon et al., 2020; Zhao et al., 2020; Eisma et al., 2024). Our data showed that the proposed One-GMM method yielded to DSC of 0.82 compared with an FS of 0.25 and the previous Two-GMM of 0.66. Although we have not done a comparison with other more sophisticated deep learning-based methods, one previous study has reported DSC 0.72 in a cohort of 98 participants aged between 21 and 89 years (Eisma et al., 2024) using fully connected U-Net and T1w, T2w, and TFLAIR images; another study reported DSC 0.72 in multiple sclerosis patients (Yazdan Panah et al., 2023) using a two-step 3D U-Net and T1w. Our proposed One-GMM method is lightweight and highly adaptive to larger datasets across vendors and study sites. This is especially valuable in AD patients, where ventricular enlargement can mislead models that rely solely on intensity gradients. We compared our method with the previous Two-GMM model mainly because both of them are GMM based and easy and fast to implement for wide clinical applications. It might be ideal to compare the proposed method with more advanced deep learning-based methods. We did not compare our results using deep learning methods, either because the model has no open-source code (Yazdan Panah et al., 2023) or because the model used different data, such as all of T1w, T2w, and T2-FLAIR (Eisma et al., 2024), as we do not have T2w in our dataset. By guiding the model with spatial priors, we reduce false-positives and improve boundary delineation as estimated by DSC, HD95, and VD%. Our results indicate that the performance of our proposed method has no diagnostic group differences within the AD continuum. In contrast, FS segmentation has a higher DSC in AD than the CN group, which is not ideal in clinical applications, as it introduces diagnostic group bias and might introduce bias to the follow-up analysis. In terms of the computational burden, the proposed method is fully automatic and utilizes the outputs of the standard FreeSurfer reconstruction, which is a typical step in current medical imaging research, followed by a light-weight subject-specific GMM processing. With the proposed region-informed segmentation, the One-GMM method takes less than 10 s per subject. Additionally, while our approach utilized FS reconstruction for LV + CP priors, any other image processing pipeline with accurate LV + CP segmentation could also be used with the proposed method.

## 5 Limitations

While our model demonstrates robust performance, this study has several limitations. First, the sample size in this study is relatively small, and the number of subjects in each diagnostic group is not balanced. This might be the reason for the lack of diagnostic difference in Cp volume in our results. Future work should validate the proposed model on a larger cohort and perform a comprehensive clinical analysis using segmented CP volume. Second, the model may introduce bias or limit generalizability to scans with atypical anatomy. We have noticed that for subjects with huge LV, the FS reconstruction of LV can be inaccurate, which might lead to an error in the CP segmentation using the proposed model. This limitation can be overcome by carefully checking the quality of FS reconstruction and editing the FS mask appropriately, or using robust LV segmentation methods. Third, the proposed One-GMM method uses initialization of the mean and standard deviation of the data, which were generated empirically. Although the mean and standard deviation are only initial guesses for those two parameters to fit into the GMM model, they might affect the segmentation results for general datasets. For each case in our dataset, the final value for each Gaussian component after segmentation is not the same as the given initial guesses. We have successfully applied these parameters to our other datasets acquired at different scanners from different vendors with the same initial guesses. We recommend that the user of the proposed method test several cases to adapt the initial mean and standard deviation parameters before applying it to larger datasets. Moreover, a soft clustering cut might be used in GMM to assign voxels to CP in future work to potentially improve the current method. In the future, a multisite data cross-validation using the proposed method and a longitudinal study of CP volume change, as well as clinical implications, might help to validate the model in clinical settings.

## 6 Conclusion

Incorporating region-based anatomical priors into a Gaussian Mixture Model significantly improves choroid plexus segmentation accuracy in healthy and Alzheimer’s disease populations. Our data show that CP volume increases with age within the CN group, but not for CP volume from the FS-based method. Our region-informed approach offers a practical and scalable solution for large-scale neuroimaging studies seeking to evaluate the role of CP in disease pathophysiology and to utilize CP structure as a biomarker of disease activity and progression.

## Data Availability

The data that support the findings of this study are available on request from the corresponding authors. The code of the GMM model in this study to generate results was written in MATLAB and is available at https://github.com/liangdongzhou/ChoroidPlexusSegmentation.
